# Federated learning for teacher data privacy protection: a study in the context of the PIPL

**DOI:** 10.3389/fdata.2026.1681382

**Published:** 2026-02-09

**Authors:** Shanwei Chen, Xiu Zhi Qi, Xue Hui Han, Zhao Chen Fan, Le Le Wang

**Affiliations:** 1 College of Education, Baoji University of Arts and Sciences, Baoji, China; 2 Academy of Fine Arts, Baoji University of Arts and Sciences, Baoji, China

**Keywords:** compliance, differential privacy, federated learning, personal information protection law, teacher data protection

## Abstract

**Background:**

The Personal Information Protection Law (PIPL) in China imposes strict requirements on personal data handling, particularly in educational contexts where teacher data privacy is critical. Traditional centralized machine learning approaches pose significant risks of data breaches and non-compliance. Federated Learning (FL) offers a promising decentralized alternative by enabling collaborative model training without sharing raw data.

**Methods:**

This study combines quantitative simulations and qualitative compliance analysis to evaluate FL frameworks under PIPL principles, with a focus on Differential Privacy as the primary empirically validated mechanism for noise addition and privacy guarantee. Other techniques, such as Secure Multi-Party Computation (SMC), are analyzed theoretically for their alignment with PIPL requirements like data minimization, anonymization, and encrypted transmission.

**Results:**

Experimental simulations demonstrate that FL effectively reduces data breach risks compared to centralized methods. It achieves principle-level compliance with PIPL through local data processing, differential privacy mechanisms, and secure aggregation, leading to improved privacy preservation while maintaining model performance.

**Conclusion:**

FL conceptually supports teacher data privacy protection under the PIPL framework. This study proposes a tailored compliance framework that integrates FL with privacy-enhancing technologies, offering theoretical foundations and practical recommendations for educational institutions and technology implementers to deploy privacy-preserving machine learning solutions.

## 1 Introduction

In the era of data-driven innovation, the rapid development of information technology and the widespread application of artificial intelligence (AI) and machine learning (ML) in fields such as education, healthcare, and finance have profoundly transformed society. National policies and regulations, including the Personal Information Protection Law (PIPL), Cybersecurity Law, and Data Security Law, have imposed stringent requirements on data processing and privacy protection, signifying China's gradual establishment of a comprehensive legal compliance framework for data security (Bakare et al., 2024). In particular, the PIPL, which came into effect in 2021, clearly defines compliance requirements for the collection, storage, processing, transmission, and use of personal information, providing a clear legal foundation for cross-domain data applications. However, as these regulations are implemented, the challenge of achieving effective data utilization while ensuring privacy protection remains a critical issue for both technological and legal domains (Jiang et al., 2024).

Traditional centralized data processing models, characterized by the centralized storage and computation of data, face significant risks of privacy breaches. The lack of transparency further undermines user trust, exacerbating compliance difficulties (Seeman and Susser, 2024). To address these challenges, Federated Learning (FL) has emerged as a decentralized ML paradigm. Since its introduction by Google in 2016, FL has gained recognition as an effective technological approach to address the challenges posed by privacy protection regulations due to its inherent advantages in safeguarding data privacy.

The core advantage of FL lies in its ability to keep data localized, exchanging only model parameters. This significantly reduces the risk of data privacy breaches. By incorporating techniques such as Differential Privacy and Secure Multi-Party Computation (SMC), FL provides robust support for legal compliance (Pustozerova and Mayer, 2020). However, studies have shown that even within FL frameworks, data privacy can still be indirectly compromised (Li et al., 2020). Thus, achieving a balance between privacy protection and regulatory compliance remains a critical issue that warrants further investigation in practice.

In light of this, the present study explores teacher data privacy protection from the compliance perspective of the PIPL, focusing on the technical implementation and legal adherence within FL frameworks. By integrating various technical methods, this research analyzes how FL can balance data privacy protection and compliance requirements and proposes a design model for protecting teacher data privacy. This study seeks to address the following key questions:

(1) How can data privacy be safeguarded within FL frameworks while ensuring compliance with the PIPL?
(2) How can Differential Privacy be integrated to enhance privacy protection in FL as the primary empirically evaluated mechanism, and what is the theoretical and compliance-oriented applicability of other privacy-enhancing technologies, such as SMC and homomorphic encryption?
(3) How can the effectiveness of FL-based privacy protection techniques be evaluated in the context of teacher data, particularly in balancing privacy protection and model performance?

Rather than serving as a stress test for real-world institutional privacy risks, the empirical evaluation in this study is intentionally designed as a controlled environment to examine the privacy–utility trade-off under a formally specified differential privacy mechanism within federated learning. Accordingly, this study does not include explicit attack-based privacy evaluations such as membership inference, gradient leakage, or reconstruction attacks, and it makes no claim of empirical robustness against specific federated learning adversarial threat models; more broadly, its scope is intentionally confined to technical design and compliance-oriented analysis under simulated conditions, and it does not purport to empirically validate user trust, institutional governance effectiveness, or pedagogical impacts in real-world educational settings.

## 2 Related research

### 2.1 Overview of federated learning

FL is an emerging distributed ML paradigm that enables multiple data sources to collaboratively train a unified model without requiring centralized data storage. Unlike traditional centralized ML methods, FL facilitates model training on local devices such as smartphones and personal computers, sharing only the trained model parameters instead of raw data. This approach minimizes the risk of data leakage and aligns with privacy protection regulations such as the PIPL.

In a typical FL workflow, a central server initializes a global model and distributes it to local devices. These devices update the model using local data and upload the resulting parameters to the central server. The server then aggregates these parameters to generate an updated global model, which is redistributed to the local devices for the next training round. This iterative process continues until a high-accuracy global model is achieved, improving both communication efficiency and security (McMahan et al., 2016).

FL is particularly well-suited for scenarios that involve highly sensitive data and decentralized data distributions, such as healthcare, education, and finance. By integrating with complementary technologies, FL demonstrates significant advantages in privacy preservation when applied to settings involving sensitive data (Rauniyar et al., 2023).

### 2.2 Applications of privacy-preserving technologies in FL

Privacy-preserving technologies are critical for ensuring the security of local data and model parameters in FL. Although data remains on local devices, various techniques are necessary to mitigate privacy leakage risks. Privacy-preserving methods include data anonymization, Differential Privacy, SMC, and homomorphic encryption. These approaches enhance FL's privacy protection capabilities from different perspectives, promoting compliance with data privacy regulations.

1. Data Anonymization

Data anonymization removes sensitive information, such as personally identifiable information, to render data unidentifiable, thereby reducing privacy risks (Song et al., 2020). However, anonymization can affect data utility and is vulnerable to linkage attacks (Majeed and Lee, 2020), where attackers may re-identify data subjects by correlating the anonymized data with other public datasets.

2. Differential Privacy

Differential Privacy introduces noise to query results to ensure that even with additional information, individual data cannot be inferred (Dwork, 2006). This method protects individual privacy and is increasingly regarded as a replacement for traditional anonymization techniques. While effective in privacy protection, excessive noise can degrade model accuracy (Zhu et al., 2022). Thus, striking a balance between privacy preservation and data utility is essential.

3. SMC

SMC enables multiple parties to collaboratively compute results without revealing their respective data. When integrated with Differential Privacy and homomorphic encryption, FL can further enhance privacy protection, ensuring that model updates do not disclose sensitive information. SMC is particularly applicable to scenarios like healthcare and finance that require cross-organizational collaboration. However, its high computational cost necessitates protocol and algorithm optimization to improve efficiency (Zhao et al., 2019).

4. Homomorphic Encryption

Homomorphic encryption allows computations to be performed on encrypted data without decrypting it, thereby ensuring data privacy. In FL, homomorphic encryption enables local model updates to be securely processed in an encrypted state, preventing data leakage. Despite its strong privacy guarantees, the high computational complexity of homomorphic encryption impacts operational efficiency (Gentry, 2009). Optimizing the computational performance of homomorphic encryption remains a key focus for future research.

Although data anonymization, SMC, and homomorphic encryption are commonly discussed as privacy-enhancing techniques in federated learning, their practical deployment in educational contexts is constrained by high computational complexity and substantial implementation challenges. For instance, SMC requires the optimization of multi-party protocols, while homomorphic encryption is plagued by efficiency bottlenecks. Given that differential privacy has been widely proven to effectively balance privacy preservation and model performance in FL scenarios, and that the current deployment conditions in educational institutions are better suited to differential privacy mechanisms with relatively low computational overhead, we have selected Differential Privacy as the primary subject of empirical validation in this study.

It should be emphasized that, despite being discussed within the same section, Differential Privacy is the only privacy-enhancing technology empirically implemented and evaluated in this study. SMC and homomorphic encryption are included solely to provide conceptual completeness and compliance-oriented context, and no experimental claims, performance comparisons, or empirical conclusions are drawn for these techniques.

### 2.3 Compliance analysis of teacher data privacy protection

In the education sector, the processing of teachers' personal information must strictly adhere to the provisions of the PIPL. Educational institutions are required to inform data subjects of the purpose of data collection and obtain their informed consent. Data processing must comply with principles of necessity and legality, and data storage must adopt effective technical safeguards to prevent leakage or tampering (Cheng and Zhang, 2023). These requirements are not only legal obligations but also critical measures to protect teacher privacy and maintain trust.

However, protecting teacher data privacy poses several technical and managerial challenges. For instance, educational institutions must balance the conflict between privacy protection and data utilization (Filgueiras, 2024). Teacher data often serve multiple purposes, such as teaching evaluations and student feedback, and excessive data collection can in-crease privacy risks. During data sharing or outsourcing, ensuring clear accountability between data controllers and processors is also a critical issue for compliance management.

From a technical perspective, FL, Differential Privacy, and SMC provide effective tools for protecting teacher data privacy. However, their implementation faces challenges related to adaptability and compliance. For example, how can FL align with the PIPL's principles of informed consent and data minimization? How can a balance be struck between security and efficiency? Addressing these questions requires identifying optimal intersections between technological solutions and legal compliance. It should be noted that the discussion of these technologies at this stage is intended to establish a conceptual and regulatory context, rather than to imply uniform empirical validation or equivalent implementation maturity across different privacy-enhancing techniques.

Moreover, compliance with teacher data privacy protection relies not only on technical solutions but also on integrated management and regulatory measures. For instance, in the event of a data breach, educational institutions are required to respond and report promptly to ensure transparency—this is a mandatory requirement under the PIPL framework (Yunfeng, 2024). As technologies like FL are applied, achieving a balance among privacy protection, data utilization, and legal compliance has become a critical issue that demands urgent attention.

To address the current challenges in teacher data privacy protection from both technical and managerial perspectives, this study will take FL as a foundation to systematically explore the implementation of privacy-preserving technologies and compliance assurance under the guidance of the PIPL. By analyzing the adaptability of technical solutions and conducting an in-depth interpretation of legal requirements, this research aims to identify a dynamic balance between privacy protection and data utilization. Furthermore, it seeks to construct a practical compliance framework to provide educational institutions with actionable guidance for managing teacher data efficiently and securely in real-world scenarios. Within this framework, compliance-related assessments are approached qualitatively and interpretively, emphasizing structured legal–technical alignment rather than quantitative claims of compliance strength or regulatory sufficiency.

## 3 Research methods and design

### 3.1 Research methods

This study employs a mixed-methods approach, combining quantitative and qualitative analysis to explore the application of FL in teacher data privacy protection and its compliance challenges.

The quantitative analysis focuses on validating the effectiveness of FL and privacy-preserving technologies in protecting teacher data through empirical evidence. Using experimental designs, the study evaluates the performance of various privacy-preserving techniques and quantifies their efficacy in ensuring privacy and compliance. This approach ensures statistical robustness and broader applicability, thereby offering solid empirical support for the compliance capabilities of privacy-preserving technologies.

The qualitative analysis involves Policy Text Analysis to examine the legal compliance of privacy-preserving technologies in practical applications, with a particular focus on meeting the requirements of the PIPL and related regulations. The study initiates this analysis with a systematic textual interpretation of the PIPL, identifying key legal provisions applicable to FL and related technologies. Special attention is given to core requirements such as data minimization, informed consent, and data transmission security. Through keyword extraction and Compliance Matching techniques, the alignment between privacy technologies and legal mandates is quantified, facilitating an assessment of both applicability and potential implementation barriers.

Ultimately, this research develops a compliance analysis framework for privacy-preserving technologies based on the PIPL, providing theoretical insights and practical guidance for ensuring the legality and effectiveness of these technologies.

### 3.2 Data sources

This study utilizes two distinct, non-overlapping public datasets. This approach ensures that the quantitative analysis of FL-based privacy protection technologies is not limited to a single data context, thereby improving the reliability and applicability of the study's findings. These datasets are selected to support reproducible and ethically compliant experimentation, rather than to emulate the full spectrum of privacy risks present in operational institutional systems, such as re-identification, linkage attacks, or governance failures.

#### 3.2.1 Teaching and learning international survey (TALIS) 2018 dataset

1. **Provenance**

The TALIS 2018 dataset is a publicly available educational survey developed by the OECD, covering 48 participating economies, including 36 OECD member countries and 12 partner economies. It can be accessed via OECD's open data portal. Accordingly, the TALIS dataset is used as a low-risk proxy to examine algorithmic behavior and privacy–utility dynamics under differential privacy, rather than as a surrogate for real institutional teacher data.

2. **Core Characteristics**
  (1) Macro-level, cross-national teacher data, including job performance, teaching evaluation, professional development, and academic achievement indicators.
  (2) Stratified sampling ensures representation across educational stages and school types.
  (3) All data are pre-anonymized by OECD; no personally identifiable information is accessible.

3. **Usage Compliance**

Access and use of the dataset were conducted within the approved academic research period, following institutional ethical guidelines and fully complying with China's PIPL.

#### 3.2.2 Kaggle open educational dataset

1. **Provenance**

The second dataset is sourced from Kaggle (https://www.kaggle.com/datasets), contributed by a research team from the University of Massachusetts Amherst. It provides teacher-level, school-specific data complementing the TALIS 2018 dataset. Similarly, this dataset does not represent operational educational information systems, but serves as a benchmark dataset for controlled evaluation of federated learning performance under differential privacy constraints.

2. **Core Characteristics**
  (1) Micro-level indicators of in-class behavior, daily work performance, and school-based evaluation results.
  (2) Covers 18,500+ anonymized teacher records across multiple regions.
  (3) Pre-anonymized by the data provider; no personally identifiable information is included.

3. **Usage Compliance**

All data usage adheres to PIPL requirements and Kaggle's license terms, ensuring ethical and legal handling.

### 3.3 Quantitative analysis design

The quantitative analysis in this study primarily focuses on Differential Privacy FL and its adaptive noise variant, compared against traditional centralized learning. This is because Differential Privacy is currently the most mature and computationally feasible approach in FL applications, while empirical comparisons of other techniques such as SMC and homomorphic encryption exceed the resource scope of this study; their compliance will be supplemented through policy compliance evaluation in the qualitative analysis.

The quantitative analysis primarily addresses the first and second research questions proposed in the introduction of this study. It integrates FL and Differential Privacy techniques to protect teacher data privacy while ensuring compliance.

In the FL framework, data remains on local devices, thereby avoiding centralized storage and transmission. Each client performs model training locally and sends gradient updates to a central server for aggregation. The central server consolidates updates from all clients and sends the global model back to them, completing the training iteration.

To further enhance data privacy, Differential Privacy is applied during each model update. By adding Gaussian noise to the gradients, sensitive information is protected throughout the training process. The noise level is adaptively tuned based on both data sensitivity and pre-defined privacy budgets, ensuring a balance between privacy protection and minimal impact on model performance. After calculating local gradient updates, each client adds noise to the gradients and sends the noise-added updates to the central server for differentially private aggregation.

The proposed mechanism follows the standard differential privacy definition (Dwork and Roth, 2014). Specifically, we employ the Gaussian mechanism for DP-SGD in a federated setting, with gradient clipping (norm bound *C* = 1.0 ) applied before noise addition on each client. Gaussian noise $\sim N\left(0,\sigma^{2}I\right)$ is added to the clipped gradients, where the noise scale σ is calibrated to achieve a per-round privacy budget of ε = 1.0 and δ = 10^−5^. The adaptive adjustment dynamically modulates σ within this budget based on observed gradient norms to optimize utility. Privacy composition across *T* = 100 communication rounds is accounted for using the moments accountant (Abadi et al., 2016), yielding a total privacy budget of approximately ε ≈ 8.0 with δ = 10^− 5^.

Let *k* represent a client, where each client trains the model using its local dataset. The categorical cross-entropy loss function is adopted as the local loss function for the multi-class teacher performance classification task (five categories). The local loss function is defined as:


$$L_{k}\left(\theta_{k}\right)=-\frac{1}{n_{k}}\sum_{i=1}^{n_{k}}\sum_{c=1}^{C}y_{i,k,c}\log\left({\hat{y}}_{i,k,c}\right)$$ (1)

Here, *C* = 5 is the number of classes, *y*_*i, k, c*_∈{0, 1} is a binary indicator, and ${\hat{y}}_{i,k,c}=f_{\theta_{k}}^{\left(c\right)}\left(x_{i,k}\right)$ is the model's predicted probability for class *c* on input *x*_*i, k*_. The ground truth labels are one-hot encoded across the five performance categories.

The local training results from each client are transmitted to the central server through model aggregation.

The clients send their noisy local model updates θ^(*t*+1)^, which is computed as in Equation 4, to the central server. The global model is then updated using standard differentially private federated averaging:


$$\begin{array}{l} \theta^{\left(t+1\right)}=\overset{K}{\underset{k=1}{\sum }}\frac{n_{k}}{n}\theta_{k}^{\left(t+1\right)} \end{array}$$ (2)

This follows the DP-FedAvg mechanism (McMahan et al., 2018), where privacy is ensured entirely through client-side gradient clipping and Gaussian noise addition, followed by weighted server-side averaging without additional noise.

To incorporate privacy protection during the training on each client, noise is added during the local training phase through an adaptive noise mechanism, resulting in noisy gradient updates:


$$\begin{array}{l} \nabla {\tilde{L}}_{k}\left(\theta_{k}\right)=\text{clip}\left(\nabla L_{k}\left(\theta_{k}\right),C\right)+\epsilon_{k} \end{array}$$ (3)

where ϵ_*k*_ is the dynamically adjusted noise variance.

The clients then send the noisy updates to the central server, and the update formula is as follows:


$$\begin{array}{l} \theta_{k}^{\left(t+1\right)}=\theta_{k}^{\left(t\right)}-\eta •\nabla {\tilde{L}}_{k}\left(\theta_{k}\right) \end{array}$$ (4)

The central server performs a weighted average of the noisy model updates sent by the clients without introducing any additional noise, as shown in Equation 2. The updated global model is then redistributed to the clients for the next round of training. This process iterates until convergence after the specified number of communication rounds.

To quantify the uncertainty in the model's predictions, this study introduces the Average Information Entropy as a complementary metric to assess the balance of the prediction distribution in a multi-class classification task, as shown below.


$$\begin{array}{l} avg\text{\_}entropy=-\frac{1}{N}\overset{N}{\underset{i=1}{\sum }}\overset{C}{\underset{c=1}{\sum }}p_{i,c}\log p_{i,c} \end{array}$$ (5)

Where *N* represents the total number of samples, *C* denotes the total number of categories, and *p*_*i, c*_ is the predicted probability that the *i*-th sample belongs to the *c*-th category.

A lower average information entropy indicates more confident model predictions, whereas a higher entropy suggests greater uncertainty in the prediction distribution. Although higher entropy can serve as a complementary indicator of the impact of noise addition, such as potentially reducing overfitting, it does not constitute a formal privacy guarantee and may vary independently of actual vulnerability to inference attacks. The primary privacy protection in this study is provided by the differential privacy mechanism. Entropy is reported here as an auxiliary metric to illustrate model calibration and prediction balance across the five performance categories: Excellent, Good, Satisfactory, Needs Improvement, and Failing. Accordingly, average information entropy is treated strictly as a model-side diagnostic of prediction dispersion and calibration under noise, rather than as evidence of privacy protection or leakage resistance.

### 3.4 Qualitative analysis design

This section primarily addresses the second and third questions raised in the introduction. Through qualitative analysis, the challenges of federal learning and privacy protection technologies in terms of legal compliance are explored. The primary method used is Policy Text Analysis, combined with keyword extraction and Compliance Matching analysis.

These methods are employed as qualitative and interpretive tools to structure legal–technical alignment, rather than as quantitative instruments for measuring compliance strength or regulatory sufficiency.

The qualitative analysis focuses on two main aspects: first, through Policy Text Analysis and Compliance Matching analysis, the alignment between the core legal requirements of the PIPL and federal learning technology is clarified; second, based on the application scenarios in the education sector, a structured, scenario-based qualitative examination of the potential compliance alignment of privacy protection models in real-world settings is conducted.

Policy Text Analysis of PIPL and related laws is conducted to identify legal requirements relevant to federated learning and privacy protection technologies. The analysis focuses on key provisions including data minimization, informed consent, data transmission security, data anonymization and de-identification, as well as compliance auditing and monitoring. These provisions are primarily drawn from the first two chapters of the PIPL, which include General Provisions and Rules for Processing Personal Information, as they establish the foundational principles most directly aligned with privacy-preserving technologies. Through regularization and keyword extraction, representative requirements in PIPL that align with privacy protection technologies are distilled, and their applicability and challenges in technical implementation are examined. Additionally, based on the envisioned application in educational settings, potential implementation pathways for federated learning models within educational institutions are proposed. The analysis includes how privacy protection measures can meet PIPL's requirements in areas such as data minimization, noise addition, and cross-institutional data collaboration.

It should be noted that the extraction of keywords and identification of representative requirements in this study are primarily based on the first two chapters of the PIPL. This is because these chapters encapsulate the most fundamental and universally applicable principles governing the processing of personal information. However, word frequency statistics serve solely as an auxiliary reference metric and cannot be equated with legal significance in a comprehensive sense. Moreover, the analysis has not been extended to subsequent chapters, which cover such areas as the cross-border transfer of personal information, the rights of personal information subjects, the performance of personal information protection obligations, and legal liabilities. As a result, this study may not have encompassed those more operationally binding obligations involved in practical deployment scenarios, including personal information protection impact assessments, specific rules for cross-border data transfers, and law enforcement mechanisms. The six selected requirements are intended only as representative principles within the focus of this study, rather than an exhaustive or core set of obligations prescribed by PIPL.

Chapters 1 and 2 articulate general principles and foundational rules for personal information processing that are most directly translatable into system-level design considerations and technical mechanisms within privacy-preserving federated learning frameworks.

Later chapters of the PIPL primarily address procedural, institutional, and enforcement-oriented obligations, such as personal information protection impact assessments, cross-border data transfer mechanisms, rights exercise procedures, accountability structures, and legal liabilities, which depend heavily on organizational context, regulatory practice, and governance arrangements. Such obligations cannot be meaningfully operationalized or evaluated through keyword-based textual analysis or technical feature mapping alone and therefore fall outside the methodological scope of the present qualitative analysis.

Regularization methods are employed to extract and clean keywords from the PIPL legal text, ensuring consistent formatting and high accuracy for subsequent statistical analysis. This process is achieved through regular expressions, including the removal of irrelevant punctuation and special characters, synonym normalization, and handling of word variations, ensuring the consistency of the textual data. The formula is as follows:


$$\begin{array}{l} \text{matches}=\overset{n}{\underset{i=1}{\sum }}\text{count }\left(T,\text{ normalize}\left(k_{i}\right)\right) \end{array}$$ (6)

In this context, “*matches*” refers to the total number of matches for all normalized keywords in the text *T*, while *n* represents the total number of predefined keywords in the keyword database, “*count*(*T, normalize*(*k*_*i*_))” refers to the frequency of the appearance of the normalized keyword *k*_*i*_ in the text *T*, and “*normalize*(*k*_*i*_)” represents the normalization process applied to the keyword *k*_*i*_.

Additionally, a Compliance Matching method is employed to quantify the alignment between privacy protection technologies and PIPL compliance requirements. This approach assigns weights to each privacy technology based on its perceived contribution and calculates compatibility scores with corresponding PIPL requirements, yielding a Compliance Matching Score (CMS). It should be noted that this scoring is based on the researchers' subjective interpretation and mapping of technical features to legal provisions, rather than rigorous independent auditing or legal assessment. Higher CMS values indicate greater theoretical alignment with the requirement. In this study, a CMS of 1.0 suggests high alignment of the proposed framework with the six core PIPL requirements under the selected mapping but serves only as a qualitative auxiliary metric to illustrate potential compliance, not as proof of full legal conformance. Such scores should be interpreted as descriptive artifacts of the mapping process itself, rather than as comparative indicators or evaluative benchmarks across systems or institutions.

The specific formula is as follows:


$$\begin{array}{l} \text{CMS}=\overset{n}{\underset{i=1}{\sum }}W_{i}\times C_{i} \end{array}$$ (7)

In this formula, *W*_*i*_ represents the weight of the privacy protection technology in meeting the PIPL requirements, and *C*_*i*_ is the compatibility score between the technology and the corresponding PIPL requirement.

**Figure 1:**
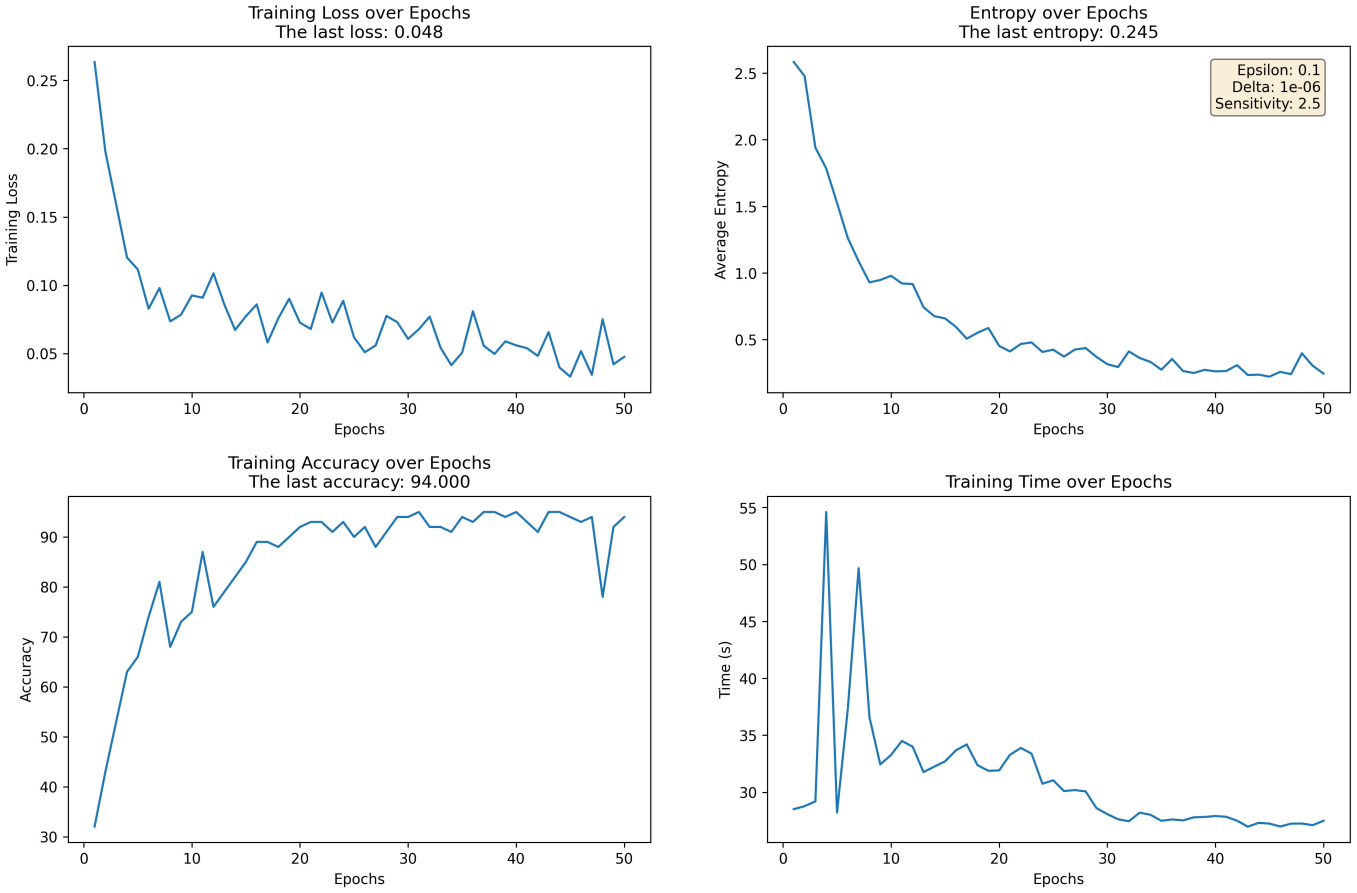
Training dynamics of FL with adaptive noise, showing trends in loss, entropy, accuracy, and training time.

**Figure 2:**
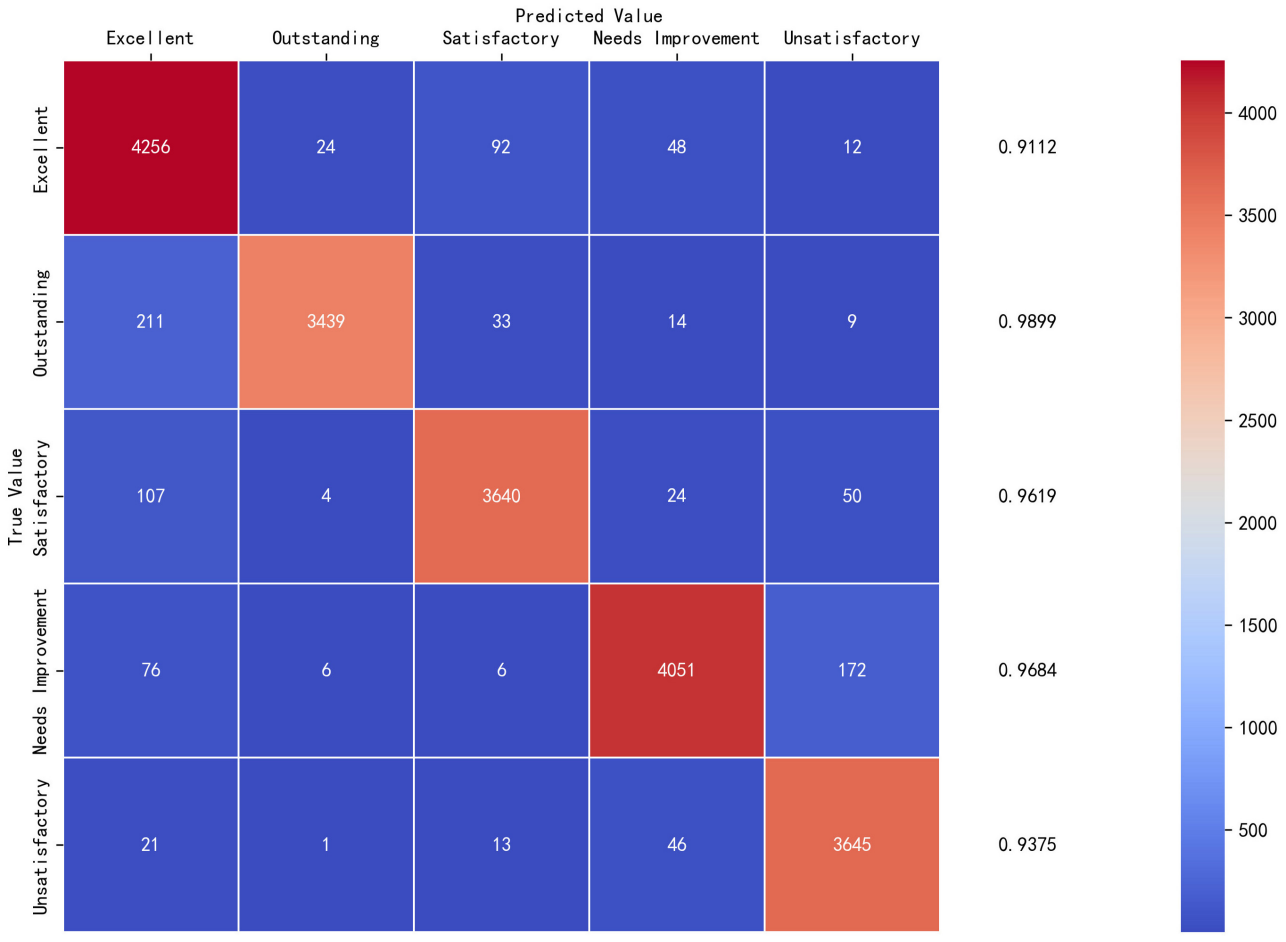
Confusion matrix of the test set with adaptive noise and differential privacy.

**Figure 3:**
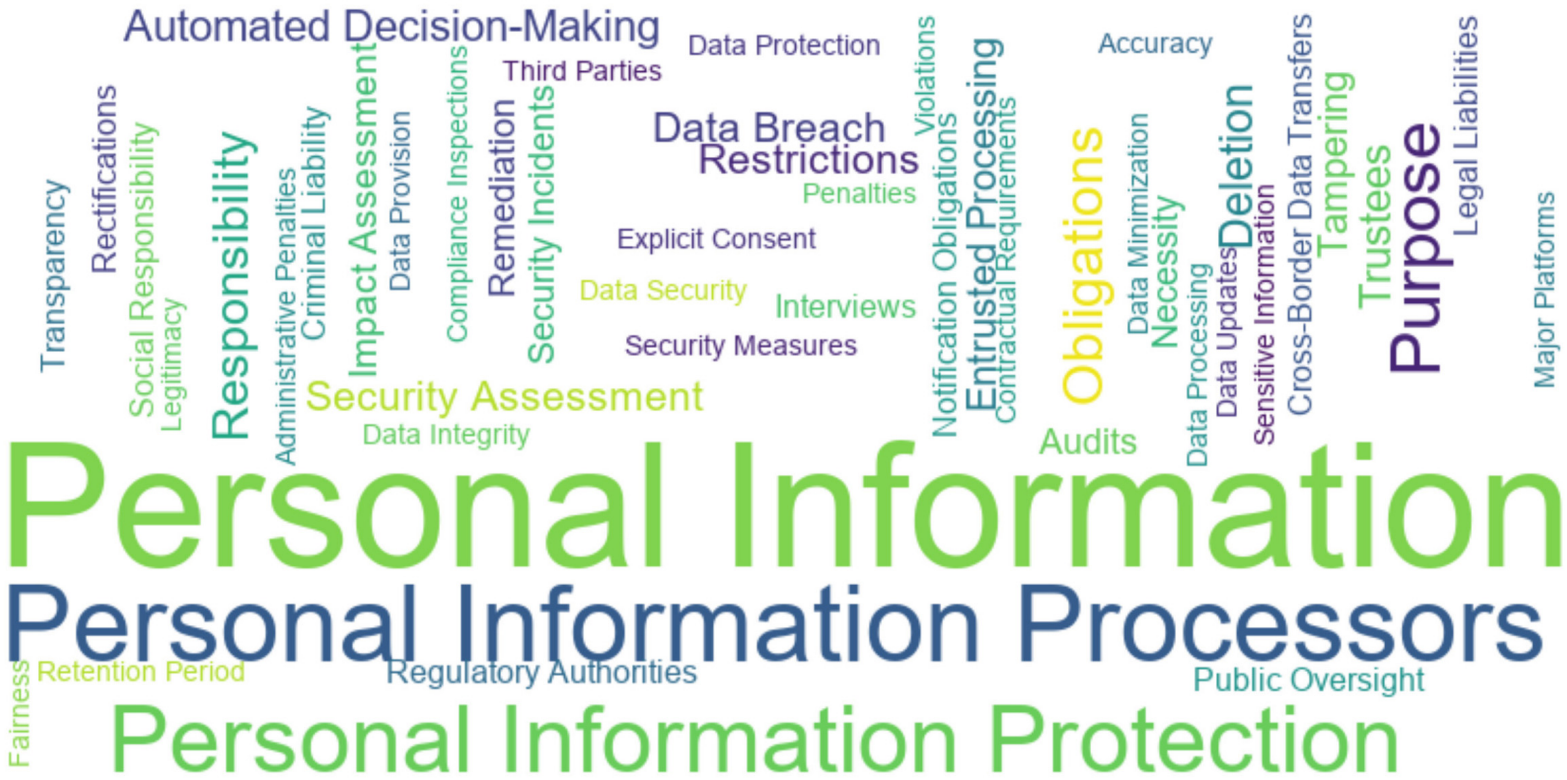
Word cloud of core compliance keywords in the PIPL.

**Figure 4:**
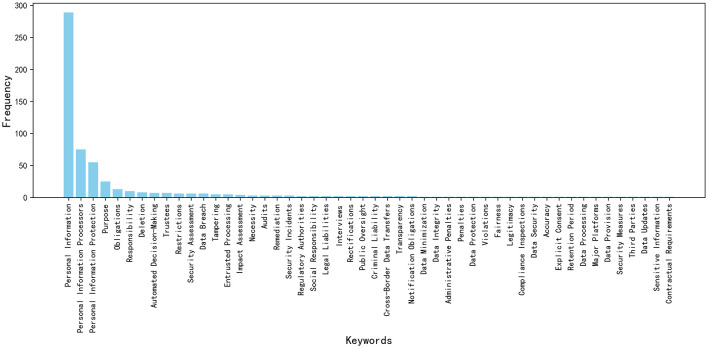
Quantitative frequency distribution of core compliance keywords in the PIPL.

**Figure 5:**
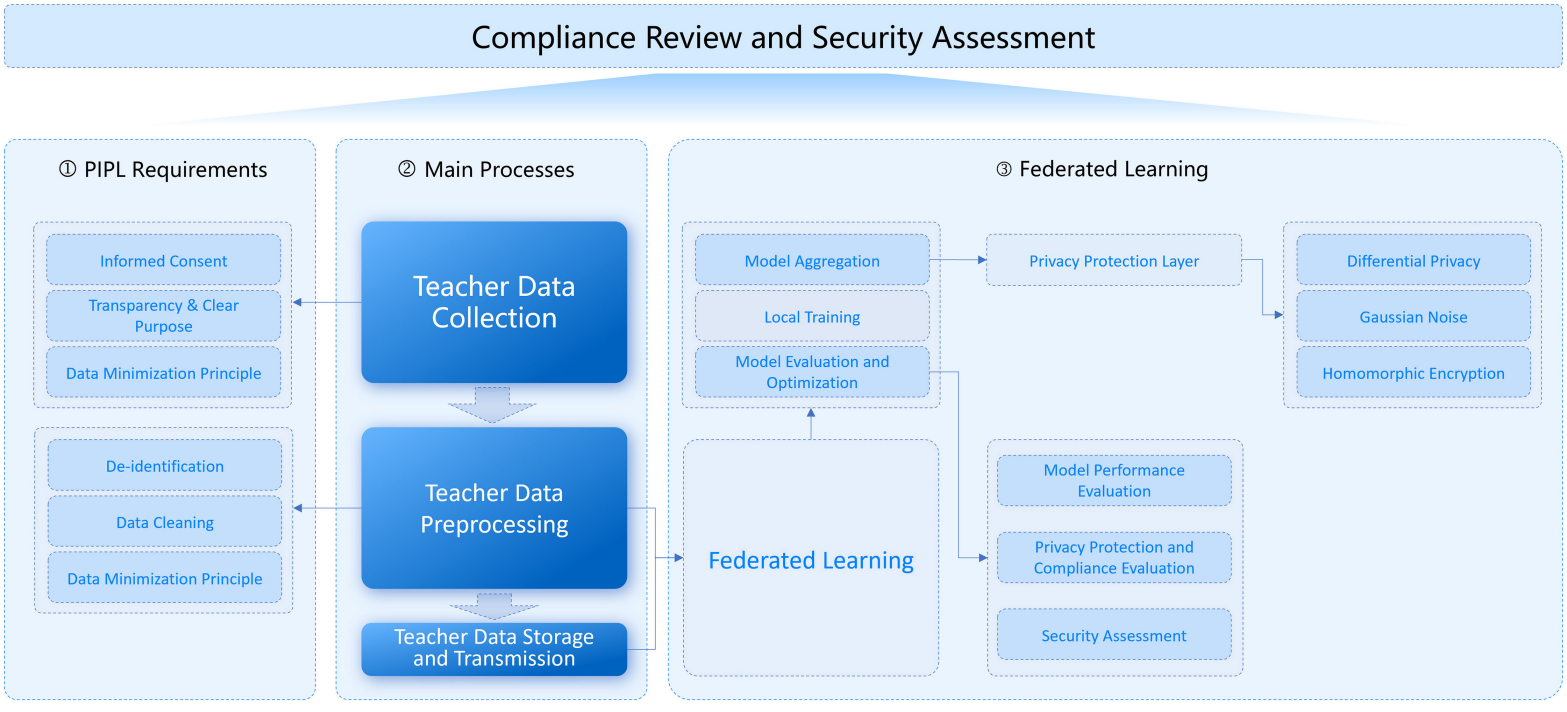
Logical architecture of the PIPL-aligned teacher data privacy protection model.

The weight (*W*_*i*_) in the Compliance Matching formula was determined using the Delphi method to ensure alignment with both legal priorities and practical applicability: First, an expert panel was convened, consisting of two legal practitioners specializing in the PIPL, two technical experts in educational data privacy, and two educational administrators—covering the “law-technology-application” dimensions critical to this study. Second, based on the six representative PIPL compliance requirements identified through policy text analysis, the panel independently scored each requirement's “compliance importance” on a 5-point scale (1 = low priority, 5 = high priority). These requirements were derived from keyword extraction and frequency statistics of PIPL chapters 1 and 2, as detailed in Tables 1–3 and Figures 1–5 in the Results section. Third, 3 rounds of anonymous feedback were conducted: after each round, the research team summarized divergent scores and provided objective references, such as keyword frequency data as detailed in the Results section, to the panel, with iterations continuing until the coefficient of variation of the scores was < 0.1, which indicates a high degree of consensus. Finally, the final consensus scores of the 6 compliance requirements were normalized to obtain *W*_*i*_ for each PIPL requirement. It should be noted that although this Delphi process followed standard procedures and sought diversity, the relatively small panel size and the absence of additional reliability metrics, such as Kendall's coefficient of concordance, may still render the weights sensitive to expert selection effects, introducing a degree of subjectivity.

**Table 1:** Performance comparison of different privacy-preserving learning approaches.

**Approach**	**Overall accuracy**	**Macro precision**	**Macro recall**	**Macro *F*1 score**	**Average information entropy**
Centralized learning	0.9934	0.9934	0.9934	0.9934	-
Differential privacy FL	0.9432	0.9452	0.9424	0.9428	0.6406
FL with adaptive noise addition	0.9516	0.9538	0.9516	0.9522	0.6472

Accordingly, the resulting weights are used solely to support internal consistency within the qualitative analysis, rather than to claim external validity or regulatory authority. They should therefore be understood as heuristic devices for structuring expert-informed discussion, rather than as statistically robust measurements or reproducible indicators of legal importance.

Finally, through qualitative coding and analysis, the extracted keywords are systematically reviewed, and key requirements in the legal text are analyzed. This process helps identify critical issues in the compliance of privacy protection technologies, ensuring that the adopted technologies can effectively align with the various PIPL requirements. Based on this, a compliance analysis logical framework for privacy protection technologies, grounded in PIPL, is constructed to provide theoretical support and compliance guidance for privacy protection measures in practical applications.

### 3.5 Experimental procedure

The experimental procedure in this study begins with data collection. The anonymized data of teachers is sourced from the open Kaggle platform, which includes information on teachers' work performance, teaching evaluations, and academic achievements.

The data processing phase takes place on local client devices, primarily involving preprocessing tasks such as data standardization, denoising, and feature extraction. Throughout this phase, strict adherence to data protection requirements is maintained, ensuring that all processing complies fully with regulations such as the PIPL.

In the model training and update phase, a FL framework is employed. Each client trains the model based on its local data and computes gradient updates. Once uploaded to the central server, these noisy gradients undergo Differential Privacy aggregation to generate the global model.

Compliance analysis focuses primarily on the legal applicability of privacy protection technologies in FL, particularly regarding their alignment with PIPL. Through Policy Text Analysis, key provisions of PIPL are thoroughly interpreted, and the legal requirements related to privacy protection technologies are identified. The analysis emphasizes areas such as data minimization, informed consent, and data transmission security, examining the compatibility and challenges between technological implementation and legal provisions to ensure that all privacy protection measures implemented during the experiment adhere to relevant laws and policies.

The evaluation of experimental results combines both quantitative and qualitative methods. In quantitative analysis, model performance, and privacy protection effectiveness (e.g., the degree of Differential Privacy assurance) are assessed using metrics such as accuracy, precision, recall, and entropy-based uncertainty measures. In the qualitative analysis, the legal compliance of privacy protection technologies is evaluated by combining Policy Text Analysis and technological implementation.

Additionally, to better simulate real-world scenarios, the experimental data distribution is modeled as non-independent and identically distributed (non-IID), reflecting the heterogeneous nature of educational data across institutions. This provides a more realistic assessment of FL's effectiveness and privacy preservation under practical conditions.

By using these complementary methods, the study ensures that technological implementation maintains a balance between privacy protection and model performance, while strictly complying with relevant laws and regulations, including the PIPL, thereby maximizing the privacy and security of teacher data.

### 3.6 Ethical considerations

This study was approved by the Ethics Committee on Human Experimentation, School of Education, Baoji University of Arts and Sciences (Approval No. BJWLXY-EDU-2024-008), effective from 23 February 2024 to 30 June 2024. The research utilized the TALIS 2018 dataset, a pre-anonymized, publicly available database from the OECD. As no identifiable personal information was accessed, the need for informed consent was waived by the ethics committee due to the retrospective nature of the study. All data processing complied with PIPL, as detailed in Section 3.2.

It is important to clarify that, under this experimental setting, no informed consent process was designed, implemented, or evaluated in practice. The waiver of informed consent reflects the use of secondary, publicly available, and pre-anonymized data, rather than the presence of any procedural consent mechanism within the proposed framework. References to “informed consent” elsewhere in this manuscript do not describe an operational consent workflow or an empirically validated ethical safeguard. Instead, they indicate only the theoretical compatibility between certain system design principles and the normative objectives commonly associated with consent requirements in personal information protection law. This study does not empirically demonstrate, simulate, or assess consent acquisition, withdrawal, or consent management processes. Any discussion of consent should be interpreted strictly at the level of conceptual system design assumptions, rather than as evidence of procedural compliance or ethical adequacy in real-world institutional deployments.

## 4 Research results

### 4.1 Quantitative analysis of experimental results

In this study, a series of simulation experiments were conducted using performance evaluation metrics for classification models, including accuracy, precision, recall, *F*1-score, and average information entropy. The confusion matrix was employed to examine the four possible prediction outcomes, including true positives (TP), true negatives (TN), false positives (FP), and false negatives (FN), thereby providing a more comprehensive understanding of model effectiveness across categories in the context of teacher data privacy protection.

It is essential to note that these results demonstrate the effectiveness of the differential privacy mechanism in mitigating information leakage and enhancing model performance within a controlled setting, but should not be interpreted as evidence of resistance to real-world privacy attacks or institutional governance risks.

To further quantify prediction uncertainty under the differential privacy setting described above, this study introduces average information entropy as a complementary metric to assess the balance of the model's prediction distribution in this multi-class classification task. By computing the entropy of predicted probabilities for each sample and averaging across all samples, the analysis evaluates how privacy-preserving measures influence prediction outcomes in the teacher performance classification task.

Taken together, it should be emphasized that no attack-based evaluation is conducted in this study. The reported performance metrics and entropy-related indicators are not intended to approximate or substitute for adversarial evaluations, such as membership inference or gradient leakage attacks. Instead, they are used to illustrate how formally defined differential privacy mechanisms regulate information dispersion and model utility under controlled conditions. While differential privacy provides an attack-agnostic upper bound on information leakage, validating resistance to specific attack strategies would require dedicated adversarial protocols and assumptions that are not adopted in the present experimental design.

Although the experiments utilize pre-anonymized public datasets, this choice was made to ensure full reproducibility, ethical compliance, and accessibility of the results. These datasets provide a controlled and reproducible baseline for evaluating the privacy-utility trade-off of the proposed mechanisms, while acknowledging that privacy risks are lower than in operational systems containing identifiable teacher data.

Figure 1 illustrates the training process of FL with adaptive noise addition, clarifying how privacy-preserving noise influences model convergence, uncertainty, accuracy, and computational stability. The four subgraphs show the trends in training loss, average information entropy, training accuracy, and training time throughout the learning process. Subgraph (1) shows the change in training loss across epochs. With the addition of noise, the loss gradually decreases and stabilizes, ultimately reaching 0.048, indicating that the model can effectively converge while preserving privacy. Subgraph (2) presents the trend of average information entropy, which gradually decreases to 0.245, indicating a reduction in prediction uncertainty under noise-perturbed training.

Subgraph (3) illustrates the trend in training accuracy. Although noise may affect accuracy initially, it increases steadily and eventually reaches 94%, indicating that privacy protection does not substantially degrade model performance. Subgraph (4) shows the training time per epoch. Although adding noise increases computational complexity, the training time becomes stable after initial fluctuations, showing that the FL framework maintains acceptable efficiency even with added privacy constraints.

Figure 2 presents the confusion matrix heatmap for the model on the test set and provides a detailed view of how well the privacy-preserving FL model distinguishes among different categories. The matrix highlights the distribution of true and predicted labels, showing that most samples fall along the diagonal, indicating strong classification performance. The relatively few off-diagonal values further suggest that the integration of adaptive noise and Differential Privacy preserves accuracy while maintaining stable prediction behavior.

The macro-average results of 10 independent experiments are presented in Table 1, which compares the performance of three approaches—Centralized Learning, Differential Privacy-integrated FL, and FL with Adaptive Noise Addition—across key metrics: Overall Accuracy, Macro Precision, Macro Recall, Macro *F*1 Score, and Average Information Entropy, which reflects prediction uncertainty and privacy protection effectiveness.

As illustrated in Table 1, while Centralized Learning achieves the highest accuracy (0.9934), it lacks privacy protection mechanisms—hence the absence of an Average Information Entropy value. In contrast, both privacy-preserving FL approaches maintain high classification performance (Overall Accuracy >0.94), demonstrating that privacy protection measures do not significantly compromise model effectiveness. Specifically, FL with Adaptive Noise Addition outperforms DP-integrated FL across all metrics (0.9516 vs. 0.9432 in Overall Accuracy, 0.9522 vs. 0.9428 in Macro *F*1 Score) and exhibits slightly higher Average Information Entropy (0.6472 vs. 0.6406). This indicates that the adaptive noise mechanism not only enhances privacy protection but also better balances privacy and model utility. Collectively, these results validate that the proposed privacy-preserving FL techniques provide robust technical support for teacher data privacy protection, enabling stable model operation and high performance while complying with privacy regulations.

### 4.2 Qualitative analysis of experimental results

#### 4.2.1 Policy text analysis

To identify the core legal requirements of the PIPL relevant to privacy-preserving technologies, this section conducts policy text analysis—through keyword extraction, text cleaning, and regularization statistics—covering all chapters of the PIPL. The analytical process involves first extracting keywords associated with personal information protection from the legal text; second, cleaning the extracted terms, such as synonym normalization, and removing irrelevant characters using regular expressions; and third, mapping keywords to corresponding legal provisions and thematic categories to clarify their regulatory implications.

Table 2 presents the keyword extraction results from the first two chapters of the PIPL, including the chapter, legal article code, related topic, regulatory description, and extracted core keywords. These keywords primarily reflect representative compliance requirements emphasized in the first two chapters, such as Explicit Consent, Data Minimization, and Data Security, which serve as the legal basis for the subsequent Compliance Matching Analysis. The text analysis provides a regulatory framework for technical compliance and offers a reference for understanding how teachers might perceive institutional safeguards, particularly in terms of fairness, transparency, and informed participation. It should be noted that these requirements were selected based on keyword extraction and frequency statistics from the first two chapters of the PIPL and serve merely as representative principles for this study, rather than representing all of the core obligations of the PIPL.

**Table 2:** Keyword extraction from the first two chapters of PIPL.

**Chapter**	**Code**	**Legal provision**	**Related topic**	**Description**	**Keywords**
Chapter 1	P1.1	Article 1	Scope of application	Defines the scope of application of the law, covering all personal information processors	Scope of application, personal information processors
	P1.2	Article 2	Definition of personal information	Establishes the definition of personal information, providing a basis for subsequent provisions	Personal information, definition
	P1.3	Article 3	Subjects of application	Specifies the organizations and individuals to whom the law applies	Subjects of application, organizations, individuals
	P1.4	Article 4	Fundamental principles of the law	Establishes the fundamental principles of personal information protection, such as legality and necessity	Legal principles, legality, necessity
	P1.5	Article 5	Data protection principles	Emphasizes principles to follow during data protection, ensuring transparency and fairness	Data protection, transparency, fairness
	P1.6	Article 6	Responsibilities for personal information processing	Outlines the responsibilities and obligations of personal information processors	Responsibilities, obligat
Chapter 2	P2.1	Article 10	Principle of legality	Personal information processing must adhere to the principles of legality, legitimacy, and necessity	Legality, legitimacy, necessity
	P2.2	Article 11	Principle of explicit consent	Processing personal information requires obtaining explicit consent from the individual	Explicit consent
	P2.3	Article 12	Principle of data minimization	Only necessary personal information should be collected, avoiding excessive data collection	Data minimization, collection
	P2.4	Article 13	Data security safeguards	Measures must be taken to protect personal data security, preventing breaches and tampering	Data security, breach, tampering
	P2.5	Article 14	Data transparency	Personal information processors must ensure transparency in data processing activities	Data transparency
	P2.6	Article 15	Data integrity	Ensure that collected and processed data is accurate and complete	Data integrity, accuracy
	P2.7	Article 16	Data storage duration	Specifies the storage period for personal information, which must not exceed what is necessary	Storage duration, time
	P2.8	Article 17	Purpose of data processing	Clarifies the purpose of data processing and ensures that it is explicit and lawful	Data processing, purpose, legality
	P2.9	Article 18	Obligation to inform	When collecting data, individuals must be informed of the purpose, methods, and other relevant details of the processing	Obligation to inform, collection, purpose
	P2.10	Article 19	Restrictions on data provision	Imposes conditions on providing personal information to third parties	Data provision, restrictions, third parties
	P2.11	Article 20	Data updating and deletion	Inaccurate data must be promptly updated, and data no longer needed should be deleted	Data updating, deletion, accuracy

Accordingly, the policy text analysis presented in this section should be interpreted as identifying a set of representative, principle-level compliance anchors rather than an exhaustive enumeration of statutory obligations under the PIPL. The absence of provisions from later chapters does not imply reduced legal relevance but instead reflects the methodological boundary of principle-oriented textual analysis adopted in this study.

Following keyword extraction and cleaning, this study conducted regularized statistical analysis to quantify the frequency of each core keyword. Word clouds and word frequency distribution charts were generated to intuitively visualize the prominence of key regulatory terms in the PIPL, such as the frequency of “data security” and “explicit consent.”

Figure 3 presents the word cloud of the extracted keywords, illustrating the importance and frequency distribution of the keywords within the text.

Figure 4 displays the frequency distribution of the keywords, helping to understand the frequency of each keyword's occurrence within the PIPL.

Through these visualizations, we gain a precise understanding of the core regulatory elements and high-frequency concepts related to data privacy protection in the PIPL. This not only verifies the legal focus on key compliance requirements but also lays a quantitative and intuitive foundation for the subsequent Compliance Matching Analysis and technical feasibility verification.

#### 4.2.2 Compliance matching analysis

To structure and qualitatively examine how the privacy protection technologies employed in this study potentially align with selected requirements of the PIPL, a Compliance Matching analysis was conducted. This analysis is presented as an interpretive organizational device within the qualitative results, rather than as an empirical measurement or evaluative assessment of regulatory compliance.

This analysis is based on the aforementioned Policy Text Analysis, which involves keyword extraction and cleaning of the PIPL text to identify the core compliance requirements within the law.

The PIPL compliance requirements focused on in this study include data minimization, informed consent, data transmission security, data anonymization and de-identification, compliance auditing and monitoring, and sensitive information protection.

To meet these compliance requirements, various privacy protection technologies were adopted, such as adaptive noise and Differential Privacy to protect sensitive information, end-to-end privacy protection mechanisms on the client side to ensure clear consent from data subjects before data processing, local data processing and de-identification to ensure data cannot be traced back to individuals, encryption during transmission, and model aggregation techniques to safeguard data during transmission. Additionally, compliance auditing and log management were implemented to ensure continuous monitoring and timely identification of compliance issues.

To further heuristically represent and organize the perceived alignment between privacy protection technologies and the PIPL compliance requirements, a Compliance Matching Formula was introduced, serving as an internal descriptive aid rather than a validated quantitative compliance metric.

This formula assigns weights to each privacy protection technology based on its relevance to its corresponding PIPL compliance requirement and calculates the degree of alignment between the technology and the law.

The assigned weights reflect expert-informed judgments used solely to structure this mapping and should not be interpreted as statistically robust parameters, generalized importance rankings, or transferable indicators across legal or institutional contexts.

Based on the integration of these technologies and legal requirements, a Compliance Matching Analysis was conducted, with results presented in Table 3. The labels reported in Table 3, such as “Compliant,” denote conceptual alignment under the study's analytical framework and do not constitute legal determinations, audit outcomes, or regulatory judgments. Furthermore, the compliance matching does not evaluate organizational governance capacity, accountability allocation, enforcement mechanisms, or institutional oversight practices, which are essential components of real-world regulatory compliance.

**Table 3:** Alignment between FL-based privacy-preserving technologies and core PIPL compliance requirements.

**PIPL requirements**	**Privacy protection technologies in the model**	**Integration approach and compliance description**	**Matching indicative alignment**
Data minimization	Adaptive noise differential privacy technology	Data is processed locally to avoid centralized storage, minimizing the risk of data exposure	Compliant
Informed consent	Client-side end-to-end privacy protection mechanism	Uses pre-anonymized public datasets, for which the Ethics Committee waived the requirement for informed consent due to the absence of identifiable personal information. Meanwhile, the framework theoretically reduces the need for frequent consent through data localization and minimization principles, which are theoretically compatible with consent-related principles under the PIPL at the level of system design assumptions, rather than constituting an implemented or verifiable informed consent process	Compliant
Data transmission security	Encrypted transmission and FL model aggregation	Encryption is used during model updates to protect the transmission process, ensuring data security during transmission	Compliant
Data anonymization and de-identification	Local data anonymization and de-identification within the FL framework	De-identification techniques are applied to teacher data to ensure it cannot be traced back to individual identities	Compliant
Compliance auditing and monitoring	Log management and regular compliance evaluations within the framework	Periodic reviews of model operation logs are conducted to ensure compliance with PIPL requirements, alongside ongoing technical audits	Compliant
Protection of sensitive information	Differential privacy and adaptive noise mechanisms	Differential Privacy is employed to ensure sensitive information cannot be identified, with noise introduced to protect data privacy	Compliant

The label “Compliant” reported in Table 3 denotes conceptual alignment under the study's analytical framework and does not constitute legal determinations, audit outcomes, or regulatory judgments.

It should be noted that the “Informed Consent” alignment reported in Table 3 reflects conceptual design compatibility only. Given that the experimental data are publicly available and informed consent was formally waived, this mapping does not represent a demonstrated consent mechanism, nor does it assess compliance with procedural consent acquisition, withdrawal, or management requirements in practice.

Based on the results of the Compliance Matching Analysis in Table 3, the six selected PIPL compliance requirements are each mapped to corresponding privacy protection mechanisms under the study's analytical framework. The resulting CMS value of 1.0 reflects complete coverage within this predefined one-to-one mapping scheme, rather than evidence of full legal compliance with the PIPL. Accordingly, this value should be read as a property of the analytical construction itself, not as a strength-of-compliance indicator.

It should be further emphasized that the compliance mappings presented here do not account for several legally binding obligations specified in later chapters of the PIPL, including personal information protection impact assessment requirements, cross-border data transfer conditions, rights exercise and redress mechanisms, and regulatory enforcement procedures. As such, the Compliance Matching Analysis illustrates conceptual alignment with selected principle-level requirements, rather than comprehensive statutory compliance with the PIPL.

To further verify the robustness of the weights in the CMS formula, a simplified sensitivity analysis was conducted: we adjusted each weight by ±10% and recalculated the CMS. Results showed that the CMS only fluctuated from the original 1.0 to a range of 0.96–1.04 (< 4%), indicating that minor variations in weights do not substantially alter the internal consistency of the mapping outcomes under the CMS framework. This sensitivity analysis supports the robustness of the descriptive mapping process itself, rather than validating compliance conclusions or external legal reliability. In particular, it does not address robustness with respect to expert selection effects, alternative weighting schemes, or independent legal interpretation.

It should be emphasized that the Compliance Matching Analysis and CMS are not intended to function as compliance certification tools or regulatory assessments. Instead, they provide a structured interpretive lens to illustrate how specific privacy-preserving mechanisms conceptually relate to selected PIPL principles within the scope of this study.

Figure 5 illustrates the logical architecture of the teacher data privacy protection model aligned with the PIPL, which is constructed based on the Compliance Matching Analysis. This figure demonstrates how privacy protection technologies are integrated with FL and the workflow for achieving full legal compliance.

The figure comprises three core components: PIPL Requirements, Main Data Processes, and FL. The PIPL Requirements section specifies the legal obligations for privacy protection, covering key principles which requirements anchor the model's compliance design; the Main Data Processes section outlines teacher data workflows, ensuring that every stage of data handling adheres to PIPL's data protection and privacy rules; and the FL section details the integration of privacy-enhancing technologies with the FL framework. This integration safeguards data privacy while preserving model performance and system security.

## 5 Discussion and conclusion

### 5.1 Application prospects of the privacy protection model in educational contexts

The privacy protection model based on FL proposed in this study, with FL combined with Differential Privacy as its core mechanism, aims to ensure the security, privacy, and compliance of teacher data within educational contexts, particularly in alignment with the PIPL. This model can be widely applied across multiple educational domains, including teacher performance evaluation systems, intelligent teaching analysis and assessment systems, teacher professional development and training platforms, as well as teacher information sharing and collaboration platforms.

The application prospects discussed below are derived from the theoretical properties of federated learning and differential privacy, rather than from direct empirical validation under real institutional privacy threat models. Accordingly, these application scenarios should be interpreted as design-oriented illustrations of how DP-based FL could be embedded within institutional workflows, rather than as empirically validated deployment outcomes or system-level guarantees. In particular, the proposed application scenarios do not incorporate empirical evaluation of teacher perceptions, trust formation, or acceptance of federated privacy-preserving systems. Nor do they assess institutional governance arrangements, accountability structures, or decision-making responsibilities associated with data processing and model deployment. As such, the application prospects should be understood as technically and legally motivated design illustrations, rather than socially or institutionally validated implementation models.

Taking an educational institution as an example, the institution seeks to improve educational quality and achieve personalized teaching guidance by analyzing data related to teachers' teaching behavior, academic achievements, and teaching effectiveness. However, this data often involves sensitive information, including personal data, work evaluations, and teaching content. The challenge lies in how to safely share and analyze this data within a compliance framework. The process based on the application framework proposed in this study is as follows:

The educational institution strictly adheres to the compliance requirements of PIPL and achieves cross-institutional data collaboration and sharing through an FL framework. In the FL process, teacher data is retained and processed on local servers, mitigating the risk of data leakage. This process complies with the stringent PIPL regulations on cross-border data transfer and external sharing, while ensuring the privacy and security of teacher data.

During the data training process, Gaussian noise is added to ensure that even if the model parameters are accessed by external observers, specific information about individual teachers cannot be inferred. Additionally, to meet PIPL's privacy protection requirements, a Differential Privacy mechanism with adaptive noise is employed. The noise standard deviation is automatically adjusted based on each data point's sensitivity to the model output, achieving an optimal balance between privacy protection and model performance. This mechanism has been empirically validated in the quantitative experiments. For other privacy-enhancing technologies such as SMC and homomorphic encryption, the framework provides theoretical extension possibilities, and their potential applications in educational scenarios await further empirical validation with improved computational resources in the future.

Furthermore, PIPL explicitly requires data processors to adhere to the principle of data minimization when collecting, using, and storing personal information. Therefore, by limiting the scope of data collection to only the necessary information related to teaching quality optimization, the collection of excessive, irrelevant sensitive data is avoided, ensuring compliance with PIPL's data minimization and purpose limitation requirements.

In practical institutional deployments involving identifiable teacher data, informed consent would need to be operationalized as an explicit and independent system component. This would include mechanisms for consent acquisition, withdrawal, record-keeping, auditability, and accountability, in accordance with the procedural requirements of the PIPL. These elements are not implemented, simulated, or evaluated in the present study, as the experimental setting relies on publicly available, pre-anonymized datasets with formally waived consent. Accordingly, consent-related considerations are positioned here as necessary conditions for future real-world adoption, rather than as technical byproducts or demonstrated features of the proposed framework.

While implementing the model, several challenges remain, primarily including the technical complexity of implementation, differences in data formats and storage systems between institutions, and detailed issues in compliance execution. In particular, when collaborating across institutions, ensuring consistency in both technical and compliance aspects among different educational entities is a critical factor for the successful application of the model, while simultaneously ensuring the model's performance and security.

### 5.2 Limitations and future research

The limitations outlined in this section reflect inherent constraints arising from the scope, design, and resources of the present study, rather than unresolved deficiencies in methodology or analysis. Several key aspects of the work, including the detailed specification of the differential privacy mechanism, the reproducibility of the federated update process, the precise role of average information entropy as an auxiliary metric, and the alignment between empirical claims and supporting evidence, have been substantially clarified, strengthened, and integrated more precisely in this revised version of the manuscript. In particular, while average information entropy is reported to characterize model uncertainty and prediction dispersion under noise addition, it serves solely as a complementary indicator of model calibration and does not support any privacy claims nor imply resistance to inference or reconstruction attacks.

First, the empirical evaluation relies on publicly available, pre-anonymized datasets. While this choice supports reproducibility, ethical compliance, and transparency, it necessarily constrains the ability to empirically stress-test real-world institutional privacy risks, such as re-identification, linkage attacks, governance failures, and institutional accountability mechanisms. These risks are more appropriately examined in operational deployments involving identifiable teacher data, institutional access controls, and real governance structures. Accordingly, this study does not claim to empirically validate resistance to such real-world privacy threats.

Second, the study focuses primarily on technical and legal-structural dimensions and does not empirically evaluate user perspectives. In particular, teachers' perceived privacy risks, trust in federated systems, and adoption intentions are not measured. While these human-centered factors are critical for real-world deployment, they fall outside the scope of the present experimental design and require dedicated user studies.

Third, broader institutional and pedagogical impacts—including data governance practices, accountability mechanisms, alignment with school policies, and potential effects on instructional workload or teaching practices—are not evaluated in the simulated environment. These dimensions depend on organizational context and policy implementation and therefore require field-based or longitudinal institutional studies.

Fourth, the present study does not empirically examine user-centered, governance-related, or pedagogical impacts of the proposed framework as an integrated socio-technical system. Specifically, teachers' perceptions of privacy risk, trust in federated learning systems, willingness to participate, and understanding of data processing practices are not evaluated. In parallel, institutional governance dimensions—such as accountability allocation, oversight mechanisms, decision authority, and compliance enforcement—are not examined under real organizational conditions. Moreover, potential pedagogical implications, including impacts on instructional workload, professional autonomy, and teaching practices, remain unexplored. User, governance, and pedagogical factors are critical determinants of trustworthiness, legitimacy, and feasibility in real-world educational deployments. However, they cannot be meaningfully assessed within a simulated, data-centric experimental setting focused on technical performance and principle-level compliance alignment. Addressing these dimensions would require interdisciplinary, field-based research involving user studies, institutional case analyses, and longitudinal evaluation of governance and pedagogical practices.

Fifth, the CMS and related qualitative analyses are intended solely as auxiliary interpretive tools. As acknowledged, the CMS employs subjective weight assignment and one-to-one mapping and is not strictly normalized, which may structurally bias scores upward and limit its use for definitive compliance assessment. Specifically, the Delphi-based weighting process is subject to expert selection effects and limited panel size, and the resulting weights should be interpreted as context-specific judgments rather than robust, reproducible, or generalizable indicators. The results derived from CMS should therefore be interpreted as illustrative rather than evaluative and do not constitute formal legal or regulatory validation. Accordingly, CMS outcomes should not be used to support claims about compliance strength, comparative regulatory advantage, or institutional readiness, and they do not replace formal legal review, regulatory assessment, or third-party compliance certification.

Sixth, the policy text analysis restricts keyword extraction primarily to the first two chapters of the PIPL and uses frequency-based indicators to identify representative compliance requirements. This approach does not capture the full spectrum of legally binding obligations articulated in later chapters of the PIPL. Consequently, neither the policy text analysis nor the downstream compliance matching results should be interpreted as evidence of comprehensive legal compliance, compliance strength, or regulatory readiness. Rather, they function as exploratory, principle-oriented analytical tools intended to support early-stage compliance reasoning and system design reflection, rather than to substitute for formal legal review, regulatory assessment, or institutional compliance certification.

Seventh, this study does not include attack-based privacy evaluations, such as membership inference attacks, gradient leakage, or reconstruction attacks. Accordingly, the empirical findings should not be interpreted as demonstrating resistance to specific federated learning threat models. In this work, privacy protection is substantiated through formally specified and composed differential privacy guarantees, which provide attack-agnostic bounds on information leakage independent of particular adversarial strategies. Incorporating explicit attack-based evaluations would require a fundamentally different experimental setup, including assumptions about attacker capabilities, auxiliary information, and threat models. Such evaluations are therefore positioned as an extension beyond the current methodological scope, rather than as missing empirical validation.

Finally, although the proposed framework is discussed in relation to informed consent principles at a theoretical level, the present study does not include any implemented, observed, or evaluated consent processes. The reliance on publicly available datasets with formally waived consent creates an unavoidable conceptual gap between normative consent requirements articulated in data protection law and the empirical behavior of the system evaluated in this study. Consequently, references to consent should not be interpreted as evidence of ethical compliance, procedural adequacy, or alignment with institutional consent practices. Addressing this gap would require future research involving operational consent workflows, user-facing consent interfaces, withdrawal and audit mechanisms, and governance arrangements within real educational institutions handling identifiable teacher data.

Future research should prioritize empirical studies in real educational institutions using operational datasets with identifiable teacher information, subject to appropriate ethical approval and governance controls. Such studies would enable realistic stress-testing of institutional privacy risks, attack-based evaluations under concrete threat models, and assessment of governance and accountability mechanisms. Equally important, future work should incorporate user-centered evaluation methods to examine teachers' trust, perceptions, and adoption intentions, as well as institutional and pedagogical impacts, through interdisciplinary collaboration across education, law, and human–computer interaction.

## Data Availability

Publicly available datasets were analyzed in this study. This data can be found here: The dataset used in this study is the Teaching and Learning International Survey (TALIS) 2018, publicly available from the OECD at: https://www.oecd.org/en/data/datasets/talis-2018-database.html. No specific accession number is assigned to this dataset, as it is openly accessible through the OECD data repository.
